# Efficacy and safety of standardized short ragweed sublingual immunotherapy tablet (SLIT-T) treatment in Canadian subjects with ragweed pollen-induced rhinitis with or without conjunctivitis

**DOI:** 10.1186/1710-1492-10-S1-A14

**Published:** 2014-03-03

**Authors:** Michael Blaiss, Peter Creticos, Jacques Hébert, Amarjot Kaur, Harold Kim, Jennifer Maloney, Harold Nelson, Hendrik Nolte, Susan Waserman

**Affiliations:** 1 University of Tennessee Health Science Center, Memphis, TN, 38163, USA; 2 Johns Hopkins University School of Medicine, Division of Allergy & Clinical Immunology, Baltimore, MD, 21205, USA; 3 Centre de Recherche Appliquée en Allergie de Québec, Québec, QC, G1V 4T3, Canada; 4 Merck, Whitehouse Station, NJ, 08889, USA; 5 Western University, London, Ontario, N6A 3K7, Canada; 6 McMaster University, Hamilton, Ontario, L8S 4L8, Canada; 7 National Jewish Health, Denver, CO, 80206, USA

## Background

Efficacy of standardized short ragweed sublingual immunotherapy tablet (SLIT-T), MK-3641 (Merck/ALK; 12 Amb a 1-U of *Ambrosia artemisiifolia*) treatment on Canadian ragweed-allergic subjects was assessed using subgroup analysis of data from 2 multinational, randomized, double-blind, placebo-controlled clinical trials designed to evaluate ragweed SLIT-T efficacy and safety in adults with ragweed pollen-induced allergic rhinitis with or without conjunctivitis (AR/C), with or without asthma.

## Methods

We conducted pooled subgroup analysis of data from 2 studies (P05234, n=784; P05233, n=565) investigating efficacy and safety of once-daily ragweed SLIT-T [1,2]. In both trials, subjects were randomized to receive ragweed SLIT-T (of multiple doses tested, 12 Amb a 1-U was found most effective and is included here) or placebo. Treatment was started approximately 16 weeks before ragweed pollen season (RS) and continued during and after RS (total, approximately 52 weeks). Subjects recorded AR/C symptoms in daily e-diaries from randomization to end of RS. During RS, subjects also recorded AR/C rescue medication use. The primary efficacy endpoint was the average total combined score (TCS), the sum of the daily symptom score (DSS) and daily medication score (DMS) during peak RS (the 15 consecutive days within RS with the highest 15-day moving average pollen count).

## Results

In the pooled study population of the 2 trials, approximately 80% of subjects were polysensitized and approximately 20% had asthma; mean age was approximately 36 years. The pooled Canadian subpopulation included 104 and 94 subjects receiving placebo and 12 Amb a 1-U SLIT-T respectively. Average pollen count for Canadian sites was approximately 100 grains/m^3^ during peak RS. Canadian subjects receiving 12 Amb a 1-U SLIT-T had a mean TCS of 5.13 over peak RS, representing a 42% reduction vs 8.90 for placebo (–3.77; 95% CI, –5.16 to –2.39). Reductions in the primary endpoint with 12 Amb a 1-U SLIT-T were supported by reductions in components DSS and DMS in the pooled population. Treatment-emergent adverse events (AEs) were reported for 80.9% and 94.5% of Canadian subjects in placebo and 12 Amb a 1-U SLIT-T groups respectively; treatment-related AEs were reported for 33.9% and 80.9% respectively. The majority of treatment-related AEs were mild, local, application-site reactions with no reports of serious treatment-related AEs or systemic allergic reactions.

## Conclusions

In pooled subgroup analysis from 2 trials, ragweed SLIT-T therapy reduced symptom and medication scores in Canadian subjects with ragweed pollen-induced AR/C with or without asthma.

## Trial registration

ClinicalTrials.gov Identifiers NCT00783198, NCT00770315
